# How Teeth Are Destroyed

**Published:** 1858-12

**Authors:** A. Wescott

**Affiliations:** Syracuse


### "HOW THE TEETH ARE DESTROYED.—BANEFUL EFFECTS OE SALERATUS AND CREAM OF TARTAR."

To the Editor of The New York Tribune, Sir: The above is the title of an article which appeared in *The Daily Tribune* of June 1st, copied from *The Portsmouth* (N. H.) *Journal.*

The author, a Mr. Baker (whether doctor, dentist, or divine, does not appear), thus commences his essay: "As a subject generally interesting and important to the public, I venture to offer some views of my own and others in regard to the articles that head this communication."

This subject is indeed an important one, in any point of view, interesting every individual in every community, when considered simply with reference to the teeth; but if the views entertained by Mr. Baker, as set forth in his article, are correct, it is preëminently so, for he not only contends that these substances which are in general use in cookery are universal tooth destroyers, but that they are also equally active in undermining the gereral health, nay, that "they are both *acrid poisons.*"

But as the article in question was evidently written to show "how the teeth are destroyed," I shall confine my review to the dental branch of the subject, leaving the rest to be settled by the various interested parties along the line—from tbe baker to the undertaker. In reference then to the "baneful effects of saleratus and cream of tartar" upon the teeth, and to which Mr. Baker unhesitatingly ascribes their premature destruction in this country, I will say that, after having carefully read his article and examined his alleged facts, I have yet to see in them the first shadow of proof in support of his remarkable theory.

Before entering, however, upon any direct examination of this subject, as it will appear in the light of *facts* and science, I beg leave to enter my protest against the author's mode of reasoning upon any scientific subject. The first item of evidence which he offers to sustain his position is the following quotation from the "popular work" of Mrs. Stowe:

"How comes it that our married ladies dwindle, fade and grow thin—their noses incline to sharpness, and their elbows to angularity," etc., at a certain period of their lives? As a matter of merest conjecture she adds, "Have not our close-heated stove-rooms something to do with it?" "Have not the immense amount of hot biscuits, hot corn-cakes, and other compounds got up with the acrid poisons of saleratus, something to do with it?"

Now, what assistance this quotation can afford Mr. Baker in establishing his theory, of dental caries, I am at a loss to determine, and I think it will puzzle him to point out any special connection between "angular elbows," "sharp noses"

and decayed teeth, unless he reverse his reasoning and make the first two consequent upon the last. But had this conjecture of Mrs Stowe's pointed directly to the teeth instead of the nose, it would still have been conjecture, and nothing else, and entitled to no more weight than any other conjecture from a lady of though great general intelligence, yet not even professing to have any knowledge of this specific topic.

Questions so purely scientific are not to be settled by the dictum or the opinion, much less by the conjecture, of persons however talented or learned in other matters, but who may be profoundly ignorant of this special department of science; and the practice, which is quite too common, of quoting the opinions of eminent persons merely because of their eminence, cannot be too strongly deprecated. The world is full and running over with senseless dogmas and prejudices which obtained their footing in this way, and which depend for the influence which they exert wholly upon their eminent origin.

The next witness introduced by Mr. Baker, speaks more directly to the point, and is no less a man than the editor of *The Boston Transcript.* He gives it as his positive *opinion* that it is cream of tartar, carbonate of soda and saleratus "which is the cause of our young men and women having decayed teeth." Will this gentleman, who is so ready with an *opinion*, inform us *how* these articles ruin teeth—whether by direct effect upon these organs, acting upon them while in the mouth, on their way to the stomach? or are they taken up by the circulating fluids, and act upon the teeth through the general system? Will he inform us why these two substances, an acid and an alkali, are always used in connection in cooking, and what is the result of this combination?

If our friend is as expert in the chemical laboratory as in the editorial chair, he will find by mixing in propor quantities cream of tartar with either carbonate of soda or saleratus, that the result will be a neutral compound, which is as inert, so far as the teeth are concerned, as so much chalk. He will find that because the component parts of a compound are acrid in their nature, it by no means follows that the compound is so, or that it partakes in the least degree of the character of either constituent. Plaster of Paris, for example, is composed of lime and sulphuric acid (oil of vitriol,) and while neither of its elements can be taken into the mouth or stomach without the most serious consequences, the compound is wholly inert and harmless. If he extend his examination to the composition and structure of the teeth themselves, he will conclude that if these articles injure the teeth at all, their "baneful effects" must be exerted directly; for finding, as he will, that the enamel is made up, for all practical purposes, solely of mineral matter, and has not the slightest circulation, he will conclude that this portion of the tooth (which is the surrounding and shield to all the rest) is neither capable of benefit nor injury through the medium of the circulation. When his examination is directed to the *bone* of the tooth, underneath the enamel, he will find the chances for reaching its substance through any vessels with which this structure is endowed not to be materially enhanced, for the circulation of the blood in the bone of a tooth is so slight as to require extraordinary circumstances to prove its existence. But even supposing it possible to communicate into the substance of the *bone* of a tooth this "baneful effect of saleratus," etc., so as to produce decay, what follows? Where would the decay be found? Evidently *on the inside* of the tooth, under the enamel. In other words, it would be an example of *internal* decay, thus involving a theory long since discarded by every intelligent dentist. If he extend his investigation still further, he will find the universal law of the system to be, that those parts which are the most vascular are the most liable to be effected through the agency of the circulation; and as the bones of the general system are a thousand times more vascular than tooth-bone, the former (upon the above theory) we would naturally expect to find, in the same ratio, more subject to disease; whereas for every case of carious bone, of the general system, we find ten thousand cases of carious teeth. In short, after he had investigated the entire subject so thoroughly as to be qualified to give an opinion which might be safely adopted by the public generally, he doubtless could and would give some better grounds for it than the only one offered by him, viz: that the French eat less saleratus than we do, and the French have better teeth; ergo, saleratus destroys our teeth. Would it not be equally good logic to say, the French drink less bad whisky than we do, and the French have sounder teeth; ergo, bad whisky is what destroys our teeth?

Mr. Baker also quotes the opinion of several doctors, to the effect that a number of students of Williamstown were made sick by eating warm biscuits and puddings "so filled with saleratus as not only to give them a yellow or burnt appearance, but also to render them bitter and nauseous to the taste." This witness further states that "after thus living for a few *months* a disease broke out, which was so severe that many believed it to be contageous." Upon this case no comment is necessary, and why it is lugged into an article whose aim is to show that saleratus destroys teeth, I shall leave the reader to conjecture. But Mr. Baker himself does not seem quite satisfied with the testimony above referred to, and hence he, as he informs us, has resorted to experiment. Now, this is doing something in the right direction—far better than to quote the opinion or conjecture of those who can not be supposed to have any special knowledge upon this difficult subject. He says, "I subjected a handful of teeth to a strong and warm solution of saleratus for about fourteen days; the consequence was they became as brittle as burnt bones." "At the same time I subjected some to a solution of cream of tartar; the consequence was not the same,but equally if not more injurious." Now, admitting the "consequences" in the above experiments to be as stated with reference to each article separately, I would ask Mr. Baker if the inference which he draws with reference to them when united, as they always are, in cooking, is correct? He has overlooked the vital fact that the two have been united; that a play of affinities has occurred during the process of raising and baking the dough that has resulted in an entire different substance from either saleratus or cream of tartar. The result, as I have already stated, is a neutral salt, perfectly harmless, so far as the teeth are concerned, and in no case would it be possible to get the separate effect of either, in cooking, unless the one or the other was used in excess—more than could be neutralized by the other. The process of raising dough by an alkali (which must always be a carbonate) and an acid is at once most simple and beautiful. The only condition necessary in the choice of an acid, when either saleratus (super-carbonate of potassa) or carbonate of soda is used, is to select one which will decompose these carbonates and unite with their base, and form with said base a a harmless salt. Sour cream, as every cook knows, is often substituted for cream of tartar. These opposite elements are so mixed with the dough that we may suppose a particle of each is resting, side by side, in every supposible point in the batch. As soon as the lump is subjected to a proper heat the decomposition and recomposition begins—each particle of acid seizing and uniting with a particle of carbonated alkali, and freeing in a gaseous form its carbonic acid, which, in its attempt to escape, forms an "air cell" in the loaf. A multiplication of these air cells thus formed gives lightness to the lump. When the bread is baked, if the saleratus and cream of tartar were used in the right proportions, there does not remain a particle of either as such. The carbonic acid of the saleratus has been expelled, and the excess of acid in the cream of tartar has united with the potassa of the saleratus and formed the neutral compound of tartrate of potassa, which is wholly inert as regards the teeth, both directly and indirectly.

Will Mr. Baker, in view of the above facts, please inform us what has become of his experiments, or rather show us their utility in reference to the articles tested, when used in cooking? He clearly having tested the wrong compound, whatever the result may have been it has no bearing whatever upon the object aimed at. The destructive effect of cream of tartar, when directly applied to the teeth, is by no means overrated by Mr. Baker. There is scarcely any acid which acts more promptly in decomposing and dissolving the enamel of a tooth, but his experiments with saleratus could not have been carefully made; certainly his record and my own, made of experiments conducted with the utmost care, do not correspond. I have yet to be convinced that salratus decomposes or dissolves the *enamel* of a tooth. Tooth-bone, which constitutes the greatest bulk of a tooth, consists (by weight) of nearly equal parts of earthy matter—phosphate and carbonate of lime—and animal matter. All alkalies have an affinity for the animal matter, but not for the earthy. The enamel, as above stated, contains hardly a trace of animal matter, and is scarcely affected by the most concentrated alkali—even caustic potash has little or no effect upon it. In the Summer of 1843, I conducted a series of experiments, assisted by Dr. Wm. Dalrymple (now of Bond street, New York), with the view to ascertain the precise effect of every substance liable to be brought in contact with the teeth, and which I have several times repeated and varied in different ways, that the result might not be deceptive, and which, instead of being confined to *two* articles, included between one and two hundred different substances. I find no such record as that made by Mr. Baker in regard to saleratus. These experiments were continued for four months, each tooth examined every forty-eight hours, and the effect upon it carefully recorded. In order to be more positive, if possible, in regard to the neutral salt, tartrate of potassa (which must not be confounded with super-tartrate of potassa, or cream of tartar, which latter has a large excess of free tartaric acid), I have, since the appearance of Mr. Baker's article in *The Tribune*, most thoroughly tested its effects upon the human teeth, and the result not only corresponds with the original record above alluded to, but fully justifies the renewal of the assertion, that this neutral salt has no effect whatever upon the teeth. Now, for the benefit of those who may have the curiosity to examine this subject, I will give a brief summary of the result of the original experiments as recorded in 1843, which is as follows:

1. Acids, both vegetable and mineral, act readily upon the bone and enamel of the teeth.

2. Alkalies do not act upon the enamel of the teeth, though the strongest of these, caustic potash, readily destroys the bone of the tooth by uniting with its animal matter.

3. Neutral salts have no effect upon the teeth, unless the acid of the salt has a stronger affinity for the lime of the tooth than for its own base; but when this is the case, the acid leaves its former base and unites with the earthy matter of the tooth, resulting in its decomposition and destruction. We have a most extraordinary example illustrating this principle in the effect of alum upon the teeth. The effect of this substance (which is a sulphate of alumina and potassa, and which contains no *free* acid) is most destructive, wholly destroying the enamel in a few days. This result can only be accounted for on the supposition that the alum is decomposed—its sulphuric acid uniting with the lime of the tooth.

4. Super salts, or those which contain a free acid, have the same effect upon the teeth as their acids would have uncombimed with a base. Cream of tartar (super-tartrate of potassa), for example, destroys the enamel of a tooth very readily, and probably with equal promptness as if the pure tartaric acid were employed, uncombined with a base. The frequent use of this acid salt in medicine, and to form acidulated drinks, renders a knowledge of its real character in reference to the teeth of great importance.

5. Vegetable substances, not acid, have no effect upon the teeth, unless by fermentation acid is formed, in which case the (acetic) acid would of course produce the same mischief as when derived from any other source.

6. Animal substances, even while in a state of confined putrefaction, act very tardily, if at all, upon either the bone or enamel. On examining the teeth subjected to such influence, the twentieth day of the experiment, no visible phenomena were presented, except a slight deposit upon the surface, of a greenish, slimy matter, somewhat resembling the green tartar often found upon the teeth in the mouth.

To give a more definite idea of the deleterious agents to which the teeth are exposed, and the consequent liability to be affected by them, I will notice the effect produced by a few of the individual substances which are more or less liable to be brought into contact with the teeth.

Acetic and citric acids so corroded the enamel in forty-eight hours that much of it was easily removed with the finger-nail. Acetic acid, or common vinegar, is not only in common use as a condiment, but is formed in the mouth whenever substances liable to fermentation are suffered to remain about the teeth for any considerable length of time.

Citric acid, or lemon juice, though less frequently brought in contact with the teeth, acts upon them still more readily.

Mallic acid, or the acid of apples, in its concentrated state also acts promptly upon the teeth.

Muriatic, sulphuric, and nitric acids, though largely diluted, soon decompose the teeth. These are in common use as tonics.

Sulphuric and nitric ethers have a similar deleterious effect, as also spirits of niter. These are common diffusable stimulants in sickness.

Raisins so corroded the enamel in twenty-four hours that its surface presented the appearance, and was of the consistency of chalk. This remarkably destructive effect of raisins upon the teeth will be readily understood by those who are familiar with the chemical and commercial history of cream of tartar. This substance exists ready formed in the juice of the grape, and is deposited as alcohol (in which it is insoluble); is formed during the process of fermentation; so that in the casks used for this purpose, a cake of crude tartar is deposited. This purified, is the cream of tartar of commerce. When the grapes are prepared as raisins, this substance is, of course, retained, and is what gives that peculiar acid flavor to this fruit in the form of raisins, and at the same time renders raisins so destructive to the teeth.

Sugar, as such, does not in the least affect the teeth; but as it promotes the fermentation of other vegetable substances which may be about, and especially between teeth, the acetic acid which would thus be formed, would prove an active source of decay.

Teeth placed in calomel (which was made of the consistency of cream by mixing it with water) came out, at the end of four months, as bright and sound as when they were placed in it; proving, most conclusively, that the direct effect of this substance, when injuriously affecting the teeth, is confined wholly and conclusively to the parts investing these organs, and hence does not act, except indirectly, in the destruction of the teeth.

From the above facts and considerations, I think every intelligent reader will conclude that the theory sought to be established by Mr. Baker, viz: that saleratus and cream of tartar, *as used in cooking*, are the chief agents in producing the proverbial rottenness of the teeth of Americans, is without the least foundation in fact, and that, on the other hand, they have little or nothing to do in producing this lamentable result—while at the same time they do point directly to many of the agencies by which the teeth are, in fact, destroyed.

With the hope that the above facts and considerations may not only do something to correct a mischievous error, but also furnish some hints which may be beneficial to the community at large, I beg leave, through your widely circulated paper, to submit them to the public.

A. Wescott, M. D., D. D. S.

*Syracuse, June,* 1858.

